# Cancer cost profiles: The Epicost estimation approach

**DOI:** 10.3389/fpubh.2022.974505

**Published:** 2022-09-21

**Authors:** Silvia Francisci, Guilia Capodaglio, Anna Gigli, Cristina Mollica, Stefano Guzzinati

**Affiliations:** ^1^National Centre for Disease Prevention and Health Promotion, National Health Institute, Rome, Italy; ^2^Screening and Health Impact Assessment Unit, Azienda Zero, Padova, Italy; ^3^Institute for Research on Population and Social Policies, National Research Council, Rome, Italy; ^4^Department of Statistical Sciences, Sapienza University of Rome, Rome, Italy; ^5^Regional Epidemiological Service, Veneto Cancer Registry (RTV), Azienda Zero, Padova, Italy

**Keywords:** cancer epidemiology, cancer cost evaluation, cancer prevalence by phase of care, cancer registry, administrative data sources

## Abstract

Sustainability of cancer burden is becoming increasingly central in the policy makers' debate, and poses a challenge for the welfare systems, due to trends towards greater intensity of healthcare service use, which imply increasing costs of cancer care. Measuring and projecting the economic burden associated with cancer and identifying effective policies for minimising its impact are important issues for healthcare systems. Scope of this paper is to illustrate a novel comprehensive approach (called Epicost) to the estimation of the economic burden of cancer, based on micro-data collected from multiple data sources. It consists of a model of cost analysis to estimate the amount of reimbursement payed by the National Health Service to health service providers (hospitals, ambulatories, pharmacies) for the expenses incurred in the diagnoses and treatments of a cohort of cancer patients; these cancer costs are estimated in various phases of the disease reflecting patients' patterns of care: initial, monitoring and final phase. The main methodological features are illustrated using a cohort of colon cancer cases from a Cancer Registry in Italy. This approach has been successfully implemented in Italy and it has been adapted to other European countries, such as Belgium, Norway and Poland in the framework of the Innovative Partnership for Action Against Cancer (iPAAC) Joint Action, sponsored by the European Commission. It is replicable in countries/regions where population-based cancer registry data is available and linkable at individual level with administrative data on costs of care.

## Introduction

Recent developments in cancer diagnosis and treatment have dramatically improved survival rates of cancer patients, especially among Western European countries. Prevalence of cancer survivorship is expected to increase because of population ageing and improved survival (1, 2), and this increasing number of cancer survivors will receive medical care along their care trajectory, starting with diagnosis and including late or lasting effects of disease and end-of-life treatments. Consequently, sustainability of cancer burden poses a great challenge for the welfare systems. Healthcare policy makers at national as well as regional level need to measure and project the economic burden associated with cancer and to identify effective policies for promoting allocative efficiency and sustainability.

From this viewpoint the core questions are:

What is the total amount of financial resources required to face healthcare needs of cancer survivors? How should these resources be allocated, taking into account patients' clinical pathway and/or the type of healthcare service provided? What are the main cost drivers? How do epidemiologic and/or demographic and/or technological changes affect the estimation of the economic burden?

Many works have addressed these issues from a life course approach, taking into account different cost components at population level (3–6). Often, however, articles concentrate on selected phases of care (7), or use selected clinical cohorts (8), or limit their analysis to specific cost components (9).

Aim of this paper is to describe a comprehensive approach: from study design to identification of cost determinants and cost estimation, using a multi-disciplinary methodology that involves epidemiological, health economic and statistical components. This approach has been developed in the Epicost study^1^ (10–12), a first attempt to measure in Italy direct medical costs sustained by the Italian National Health Service (NHS) for services provided to patients, along their disease pathway, using micro-data integrated from multiple population-based data sources.

Throughout this paper the Epicost approach is illustrated and compared to other existing methods; each sub-section provides evidence to address the above listed questions.

The paper is organised as follows: Section Study design illustrates the main features of the study design; Section Cost estimation describes methods to estimate costs; in Section An example: the case of Veneto CR an example of application is used to illustrate the study design, the data requirements and the main methodological features; finally, a general discussion and future developments are reported in Section Discussion.

## Study design

### Integrating population-based data

The Epicost approach applies to data sources referring to the whole population (population-based), and integrates information on an individual basis from two types of data sources: Cancer Registry (CR) and Healthcare Services databases. The former provides demographic and clinical information on cancer patients, the latter provide information on access to healthcare services and corresponding costs, defined as the reimbursement payed by the National Health Service to health service providers (hospitals, ambulatories, pharmacies) for the expenses incurred in the diagnoses and treatments of a patient. The two types of data sources are integrated at an individual level.

CR collects data, on an individual basis, on all cancer diagnoses that occur in the population residing in the area covered by cancer registration. The following variables are routinely collected for each patient: gender, date of birth, date of diagnosis, site of primary tumour, morphology, basis of diagnosis, vital status, date of last follow-up. Additional clinical variables about the severity of the disease and the cancer progression are collected from patients' clinical records for selected cancer sites, such as colorectal and breast.

Healthcare Services databases collect data, on an individual basis, on direct medical costs, i.e., costs associated with healthcare services that patients receive for cancer care during the entire clinical pathway, such as diagnostic examinations, hospitalisation, surgery, physician visits, radiation therapy, chemotherapy/immunotherapy, emergency room access, drugs, hospice, home care delivery, medical devices: the more data is available and linkable, the more should be used. Moreover, the reader should bear in mind that these are real (i.e. observed) costs, used in the Epicost strategy to estimate the main determinants of oncological expenditures and their amount for specific demographic and clinical patients' profiles. The services and related costs are based on well-established classification systems: for example, Diagnosis-related group (DRG)-based for inpatient costs, national or regional lists of codes for outpatient services, national or regional reference prices for pharmaceutical prescriptions.

In estimating expenditure burden we adopt a public health perspective, that is the costs considered in the study are those medical expenses sustained by healthcare infrastructures and claimed to the healthcare authority as reimbursement. Expenditures payed by the patient, such as co-payment and out-of-pocket payments, are excluded.

Management of cost reimbursement, and consequently of information collection, varies over countries. However, the Epicost approach can be applied, regardless of the reimbursement scheme, as long as information on cancer patients and costs is available and linkable at an individual level. A first attempt to export this approach to other European countries has been successfully tested within the framework of the Innovative Partnership for Action Against Cancer (iPAAC), a Joint Action funded by the European Commission under the Third Health Programme 2014–2020 with the aim at developing innovative approaches to advances in cancer control.^2^

### The study cohort

Cancer costs may refer to: (i) a longitudinal cohort of patients that are homogenous with respect to the year/period of diagnosis and are followed up for a number of years (incidence costs); in every year following diagnosis, incidence costs include only costs of surviving patients (13); (ii) a cross-sectional cohort of patients that are alive in a specific date regardless of when they were diagnosed (prevalence costs); prevalence costs provide a snapshot of the total costs delivered to cancer patients in a given calendar year (6).

Both incidence and prevalence cost indicators can be used for resource allocation, as well as policy and program planning: incidence costs are commonly used in cost-effectiveness models for decisions about specific therapies (14), whereas prevalence costs are most commonly used in quantifying the overall impact of disease on health budgets (5, 6), in monitoring resources used by patients with a similar cancer and in planning appropriate future resources (4).

The main disadvantage of a longitudinal cohort, when observing it for a long period, is that treatments and procedures evolve over the years and costs referring to the post-diagnostic phase may not reflect the more recent situation. On the other hand, a cross-sectional cohort is not homogeneous with respect to the period of diagnosis, and this might affect the distribution of cases and costs by phase of care, in the case of abrupt changes in incidence and survival over the observational period.

In the framework of the Epicost study, where the main objective is the estimation of the economic burden of cancer onto the National Health System, the cross-sectional cohort is preferable. It has been originally introduced by demographers to estimate population life expectancy at birth and has been successfully applied in cancer descriptive epidemiology to provide up-to-date estimates of cancer survival from population-based cancer registry data (3, 15–17). The maximum number of years of incidence used to identify the cross-sectional cohort depends on the data availability and on the survival features of the index cancer considered: according to a recently published study on cancer cure in Europe (18), the Time-to-Cure, defined as the number of years after cancer diagnosis when the excess mortality due to cancer becomes negligible, was less than 5 years for testis and thyroid cancer patients diagnosed below age 55, and less than 10 years for stomach, colorectal, corpus uteri and melanoma patients of all ages; for breast and prostate cancers, a small excess (5-year conditional relative survival <95%) remained for at least 15 years.

### The phase-of-care framework

Costs are not uniformly distributed along a patient's care path, and the phase-of-care (POC) framework, based on cancer patients' clinical pathway, is commonly used for estimating cancer medical costs. It consists in subdividing patient's life-time into clinically relevant periods in relation to diagnosis and disease outcomes, such as surveillance for possible recurrences, chronical conditions, death, or complete remission. In the international literature (3, 19–22) three main clinically relevant phases are typically identified:

- *Initial phase*, starting from diagnosis and characterised by diagnostic procedures and main course treatments that are mainly provided to a patient in the first months of the clinical pathway.
- *Final phase*, measured backward from death due to cancer and characterised by palliative procedures administered to the patient in the terminal status.
- *Continuing phase*, the time in between initial and final phases, mainly characterised by surveillance procedures.

Cost profiles, that describe individual average costs along time, follow a U-shape, with higher costs during the first year after diagnosis and the last year before death, and lower costs during the intermediate phase. Duration of phases is empirically attributed, accordingly.

The POC approach is applicable to either type of cohort (longitudinal or cross-sectional). In the case of a cross-sectional study cohort, each patient at the prevalence date can only belong to one phase of care and costs related to his/her treatment will be associated to that specific phase of care (10, 11, 23).

**Figure 1:**
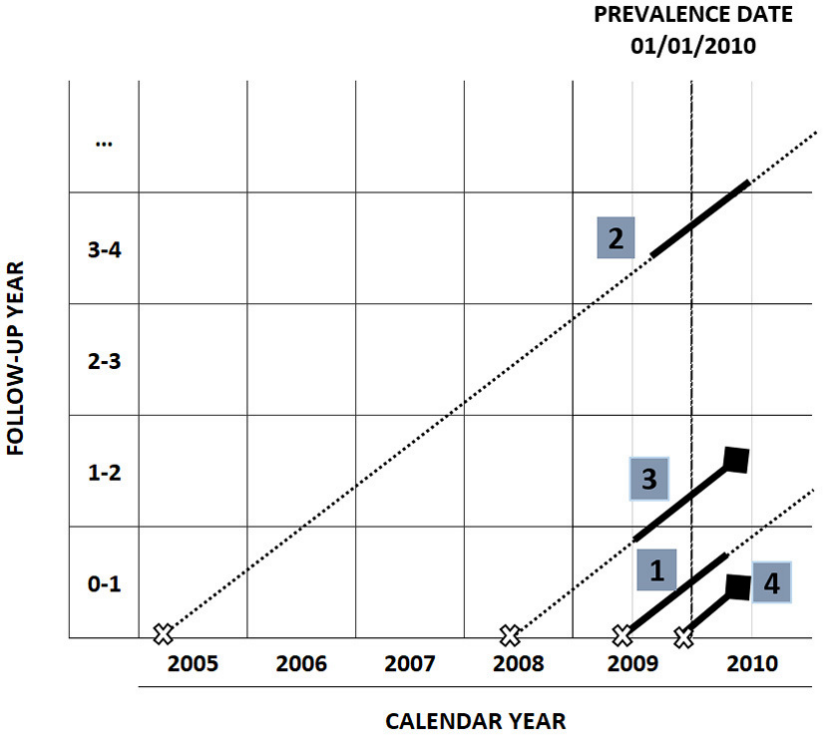
Phase of care attribution in a cross-sectional study design with prevalence date 01/01/2010.

Figure 1 shows how to attribute the phase of care, according to the cross-sectional study design, to a group of hypothetical cancer patients diagnosed before year 2010 and alive on 01/01/2010: the horizontal axis represents the calendar year and the vertical axis represents the follow-up time since diagnosis. The dashed vertical line in correspondence of January, 1st 2010 is the prevalence date. Each diagonal line represents the life trajectory of a patient after diagnosis: a cross indicates the time of diagnosis, a diamond the time of death. Phases of care are attributed to each patient according to date of diagnosis, date of last follow-up and vital status as follows:

A patient diagnosed within the 12 months prior to prevalence date, who survives at least 12 months after prevalence date, belongs to the initial phase (patient number 1).

A patient diagnosed more than 12 months before prevalence date, who survives at least 12 months after prevalence date, belongs to the continuing phase (patient number 2).

A patient who dies within 12 months from prevalence date, having survived at least 12 months after diagnosis, is attributed to the final phase (patient number 3).

In the case of a patient with an overall survival shorter than 12 months (patient number 4), the first 2 months after diagnosis are attributed to the initial phase, and the remaining months to the final phase.

A censored patient (lost to follow-up or death for causes other than cancer) is attributed to the initial or the continuing phase of care according to the date of diagnosis.

Hence, in order to assign the phase of care to each patient, the required variables are: date of diagnosis, vital status, date of last follow-up. Cause of death is also necessary to distinguish deaths due to cancer from those due to other diseases, as patients dying for causes other than cancer are censored; when information about the cause of death is not available or unreliable, all deaths occurring in the study cohort are assumed as due to cancer.

Costs are estimated in a period of 12 months around prevalence date, except for short-term survivors or censored patients in initial phase, who will contribute for less than 12 months. In Figure 1 costs attribution intervals are identified by a solid segment in the patient's life trajectory and are defined as follows:

- [diagnosis date, diagnosis date + 12 months] for patients in initial phase.
- [prevalence date – 6 months, prevalence date + 6 months] for patients in continuing phase.
- [death date – 12 months, death date] for patients in final phase.

## Cost estimation

### Total vs. incremental costs approach

There are two broad methodological approaches in cost studies: total or incremental costs (24).

The total costs approach provides estimates of the healthcare expenditure of a cohort of people diagnosed with the disease; the incremental costs approach estimates the increase in costs that is attributable solely to the presence of the disease. The incremental costs approach involves the estimation of costs of a comparison group of patients without the disease - the so-called matched control group - which are subtracted from the costs of the cohort group; the total cost approach does not require a comparison group of patients.

The incremental costs approach requires a proper matching between cases and controls, with respect to all confounding factors. This approach is typically used in studies on the cost of cancer in the United States, which are based on the linkage of SEER (Surveillance, Epidemiology, and End Result Program) and Medicare databases (20, 25): SEER contains clinical information on cancer patients, Medicare contains cost information on cancer and non-cancer patients. The incremental costs approach is also applicable when information on procedures/intervention/drugs administered is available before cancer diagnosis and patients can be used as self-controls: a comparison between the type and amount of procedures/intervention/drugs administered before (controls) and after (cases) cancer diagnosis is used to identify those procedures/drugs that are cancer-related.

The total costs approach, when restricted to expenditures directly related to the disease of interest, requires an accurate and complete identification of these expenditures. This approach, also known as attribution method (12, 25, 26), requires the expertise to identify procedures, interventions and drugs related to cancer, taking into account the classification systems used, the available clinical guidelines and the current practise. Once these lists of cancer-related procedures are selected, they can be used, for example, to estimate the cost of cancer (10, 11), or to identify and compare patterns of treatment of patients affected by a specific cancer in different countries (27).

Both approaches (estimation of total or incremental costs) have advantages and disadvantages and their choice depends on the aim of the study. When dealing with chronic diseases with multiple risk factors that are common to other pathological conditions, a satisfying degree of case/control matching might be difficult to obtain; even more so with cancer, that develops especially among elderly people, who have a number of co-morbidities. Consequently, the incremental costs approach may overestimate the costs of illness. On the other hand, the total costs approach may underestimate costs if “spill over” costs are not included. For example, the risk of bone fracture is increased in the presence of bone metastases, therefore it should be taken into account.

In the Epicost approach, the attribution method has been implemented (12) and lists of cancer-related treatments/procedures/drugs have been selected on the basis of international classification systems and can be easily applicable to other countries. We include costs related to cancer care complications (such as bone fracture) as well as costs related to generic procedures (such as electrocardiogram, or routine chest x-rays) that, being administered to cancer patients, are supposedly related to cancer care.

### Cost indicators

**Figure 2:**
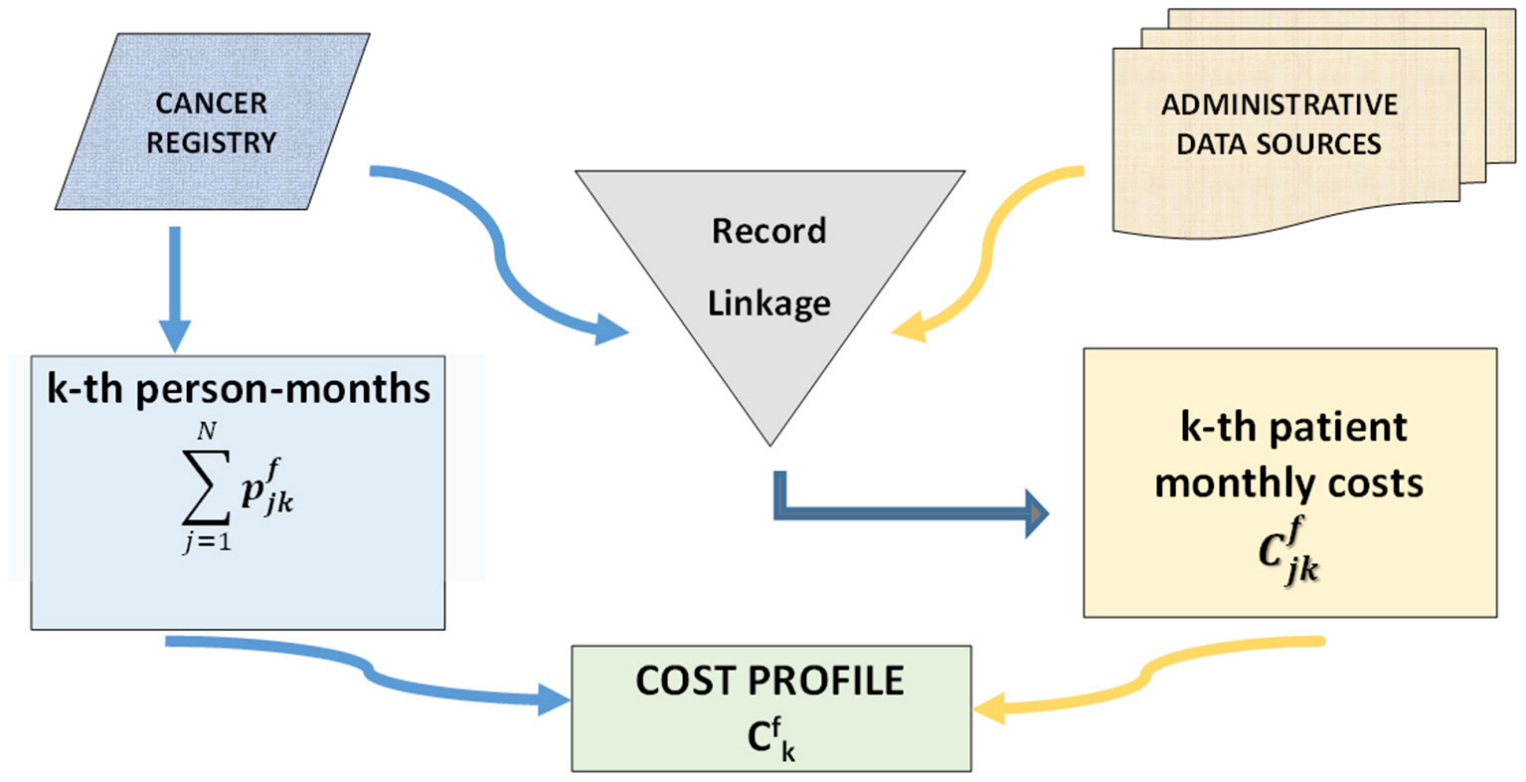
Flow-chart of the main steps of the cost estimation algorithm.

The flow chart in Figure 2 illustrates the main steps of the cost estimation algorithm: linkage of the data sources; information on prevalent cases comes from the CR; information on costs from the administrative data sources. The following indicators are computed: ${\text{p}}_{\text{jk}}^{\text{f}}$ is a binary indicator that equals 1 if patient j (j = 1,..., N) is alive in month k (k = 1,..., 12) of phase f (f = initial, continuing, final), and 0 otherwise, and it is used to compute person-months; ${\text{C}}_{\text{jk}}^{\text{f}}$ are costs of patient j in month k in phase f; patient monthly average costs ${\text{C}}_{\text{k}}^{\text{f}}$ are costs borne on average for a patient in month k of phase f, computed by dividing costs of all patients in month k and phase f by the corresponding person-months:


(1) $$\begin{array}{l} C_{k}^{f}\;=\;\frac{\sum_{j\;=\;1}^{N}C_{jk}^{f}}{\sum_{j\;=\;1}^{N}p_{jk}^{f}} \end{array}$$

The sequence of patient monthly average costs in each phase of care (${\text{C}}_{1}^{\text{initial}}$,.., C_12_^initial^, ${\text{C}}_{1}^{\text{continuing}}$,.., ${\text{C}}_{12}^{\text{continuing}}$, ${\text{C}}_{1}^{\text{final}}$,.., ${\text{C}}_{12}^{\text{final}}$) constitutes a costs profile.

**Figure 3:**
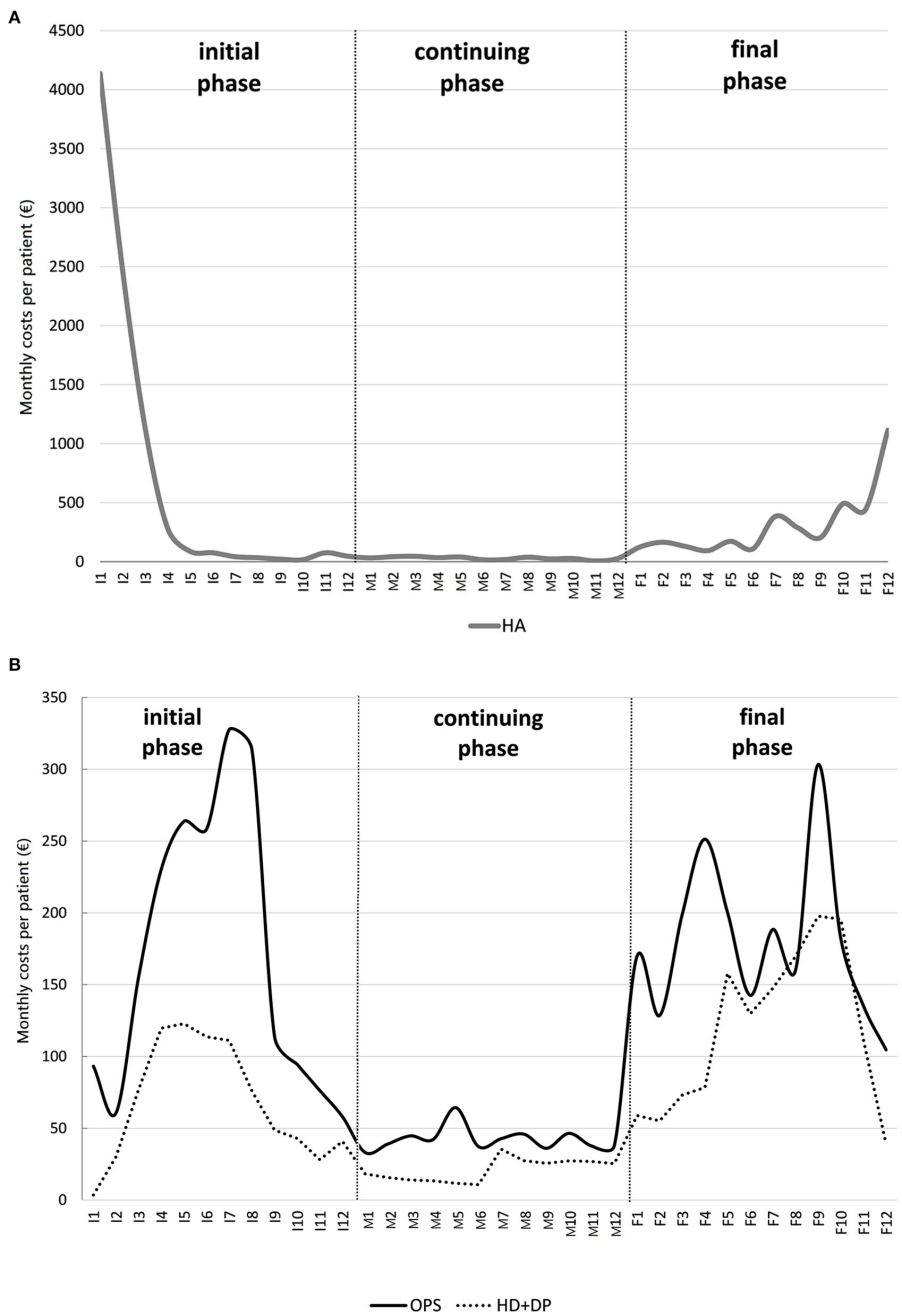
Example of a colon cancer patient cost profile due to **(A)** hospitalisation and **(B)** outpatient services and drugs (hospital drugs and drug prescriptions combined).

A cost profile plot is illustrated in Figure 3A for hospitalisation costs (HA) and Figure 3B for outpatient services costs (OPS) and drugs costs (DP+HD), for the cohort described in the example of section An example: The case of Veneto CR: the X-axis measures the time in each phase of care: I_1_,..., I_12_ indicate the 12 months of the initial phase; M_1_,..., M_12_ the 12 months of the continuing phase; F_1_,..., F_12_ the 12 months of the final phase. The Y-axis measures the monthly average cost per patient.

Patient yearly average costs in phase f, C^f^, are obtained by dividing costs of all patients in phase f over 12 months by the corresponding person-months and multiplying the ratio by 12, i.e.


(2) $$\begin{array}{l} C^{f}\;=\;\frac{{\sum^{\text{​}}}_{k\;=\;1}^{12}{\sum^{\text{​}}}_{j\;=\;1}^{N}C_{jk}^{f}}{{\sum^{\text{​}}}_{k\;=\;1}^{12}{\sum^{\text{​}}}_{j\;=\;1}^{N}p_{jk}^{f}}\;\times \;12 \end{array}$$

Within the study cohort we can identify groups of patients that are homogeneous regarding to demographic and clinical features which affect patterns of care and compute costs profiles of homogeneous groups, calculated by averaging costs over patients of the same group.

### Modelling healthcare costs

Identification of those features that most influence costs can be achieved by statistical modelling. The modelling of healthcare costs, however, can be a challenging task due to the peculiar features characterising the data distribution, that can require the use of specific statistical methods besides the most traditional approaches to obtain reliable estimates.

The most promising models can be embedded under the general framework of the regression modelling, which is a consolidated strategy to investigate the relationship between the economic burden of cancer and a set of possible predictors, typically involving patients' socio-demographic, as well as clinical conditions and management systems.

Standard statistical regression approaches, such as the normal linear model, are not suitable as the costs data are typically characterised by: (i) a remarkable positive skewness, such that the cost distribution mostly concentrates over lower values (in the surveillance phase) with a certain portion of patients undergoing more expensive treatments (at the onset of the disease and in the end-of-life), resulting in a long right tail; (ii) heteroskedasticity, which may depend on the value of one or more covariates and, hence, may have a form that can be difficult to be handled; (iii) zero costs for those patients who did not receive any healthcare service.

In order to deal with these issues a variety of strategies have been proposed in the literature (28–30). These can be broadly grouped in the following approaches: (a) data transformations, in particular the logarithmic one, which is applied in the attempt to reduce the skewness and heteroskedasticity issues and to obtain more reliable estimates from the fit of the linear regression model (31); (b) the relaxation of the normal assumption and the use of other sampling distributions for the cost outcomes within the generalised linear model (GLM) framework (32); (c) the adaptation of standard survival analysis model, such as the Cox proportional hazard model (30).

The first approach induces a retransformation bias, an issue affecting methods based on log-transformed data (33). The latter approach has been criticised, as it relies on the restrictive proportional hazards assumption, which is often violated in practise (34). GLM admits a wider range of parametric distributions for the response variable and its flexibility can be conveniently exploited to work with the total expenditure outcome on the original scale is the preferred approach in the Epicost framework.

## An example: The case of Veneto CR

The main features of the Epicost approach are illustrated using a cohort of colon cancer cases from the Veneto Cancer Registry (VCR), in the North-East of Italy.

The study cohort is retrospectively identified on the basis of the site of primary tumour (index tumour), i.e. colon (ICDO3: C18), and includes all patients that are alive on 1.1.2010 (prevalence date), and have been diagnosed with colon tumour in 20 years, spanning from 1990 to 2009 (incidence period). Individuals with multiple tumour diagnoses within 5 years from the colon cancer are excluded, as the attribution of costs to the index tumour would be uncertain.

Procedures, interventions, drugs and related costs are stored in four different sources: Hospital Admission database (HA), Outpatient Services database (OPS), Drug Prescriptions database (DP) and Hospital Drugs database (HD). Procedures and interventions are classified according to the International Classification of Diseases (ICD9-CM); drugs are classified according to the Anatomical Therapeutic Chemical Classification System (ATC).

The HA database is a collection of hospital admissions, discharges and related observed costs based on the DGR system; the OPS database provides information on outpatient services (for example, diagnostic tests and ambulatory interventions) and related observed costs derived from the Veneto regional list of codes; the DP database contains data on drugs prescribed to a patient and sold by a pharmacy and related observed costs derived from the Italian national reference price list; and the HD database contains data on high cost drugs administered to a patient during hospitalisation and related costs are negotiated by the local health authority and can vary within the same region. Data is collected on an individual basis and includes a personal identification code for the record linkage with the CR database, in order to trace all healthcare resources utilised during the patient observational time interval.

The study cohort includes 2,085 colon cancer cases alive at 1.1.2010.

Using deterministic linkage with administrative data sources, a total of 2,375 hospitalisation, 163,025 outpatient records, 4,961 hospital drugs and 118,637 pharmaceutical prescriptions were linked to the study cohort. According to the attribution method 66.5% of hospitalisation, 69% of outpatients procedures, 55% of hospital drugs and 5% of pharmaceutical prescriptions are related to colon cancer and contributed to the cost estimation.

Three sets of variables are provided and described in Table 1:

a. Socio-demographic status: gender; age class at prevalence (15–59; 60–69; 70–74; 75–84; 85–110 years).
b. Clinical condition: stage at diagnosis (from I = local/early to IV = advances/metastatic); cancer sub-site: proximal (ICDO3: C18.0–C18.4), distal (ICDO3: C18.5–C18.7), NOS (Not Otherwise Specified); years since diagnosis; Charlson Index (35) indicating the presence of comorbidities, grouped in three categories: patients with no comorbidities (Charlson Index = 0), patients with at least one comorbidity (Charlson Index > 0) and those with Charlson Index undetermined (not available, NA).
c. Fruition of medical facilities: total number of treatments received by the patient during the phase of care. This was mainly included as a control variable, since it is expected that patients receiving a higher number of treatments are associated with higher costs (36).

**Table 1:** Descriptive summaries of the cross-sectional study cohort, average patient costs by type of service, total costs of the study cohort (grand total cost), by phase of care.

**Initial phase**		**Continuing phase**		**Final phase**	
**Variable**	**Frequency (%)**	**Variable**	**Frequency (%)**	**Variable**	**Frequency (%)**
**Gender**		**Gender**		**Gender**	
Male	125 (49.8)	Male	881 (51.9)	Male	78 (57.8)
Female	126 (50.2)	Female	818 (48.1)	Female	57 (42.2)
Total	251 (100.0)	Total	1,699 (100.0)	Total	135 (100.0)
**Age class**		**Age class**		**Age class**	
[15, 60]	58 (23.0)	[15, 60]	234 (13.8)	[15, 60]	15 (11.1)
[60, 70]	80 (31.9)	[60, 70]	460 (27.1)	[60, 70]	21 (15.6)
[70, 75]	22 (8.8)	[70, 75]	263 (15.5)	[70, 75]	11 (8.1)
[75, 85]	63 (25.1)	[75, 85]	549 (32.3)	[75, 85]	47 (34.8)
[85, 110]	28 (11.2)	[85, 110]	193 (11.4)	[85, 110]	41 (30.4)
**Stage**		**Years since diagnosis**		**Years since diagnosis**	
I	95 (37.8)	(1, 2)	204 (12.0)	[0,1]	30 (22.2)
II	78 (31.1)	(2, 3)	194 (11.4)	(1, 3)	37 (27.4)
III	59 (23.5)	(3, 4)	168 (9.9)	(3, 4)	12 (8.9)
IV	19 (7.6)	(4, 8)	490 (28.8)	(4, 20)	56 (41.5)
		(8, 20)	643 (37.8)		
**Sub-site**		**Sub-site**		**Sub-site**	
Distal	124 (49.4)	Distal	564 (33.2)	Distal	38 (28.1)
Proximal	123 (49.0)	Proximal	354 (20.8)	Proximal	43 (31.9)
NOS	4 (1.6)	NOS	781 (46.0)	NOS	54 (40.0)
**Charlson Index**		**Charlson Index**		**Charlson Index**	
0	198 (78.9)	0	518 (30.5)	0	67 (49.6)
>0	43 (17.1)	>0	203 (11.9)	>0	52 (38.5)
NA	10 (4.0)	NA	978 (57.6)	NA	16 (11.9)
**Treatments/Costs**	**Mean (sd)**	**Treatments/Costs**	**Mean (sd)**	**Treatments/Costs**	**Mean (sd)**
No. treatments	47 (50.6)	No. treatments	26 (28.4)	No. treatments	64 (70.3)
HA costs	8,287 (4268.0)	HA costs	291 (1,424.4)	HA costs	3,564 (4,553.8)
OPS costs	2,064 (3,061.3)	OPS costs	399 (946.7)	OPS costs	2,032 (3,272.4)
DP + HD costs	811 (2,695.5)	DP + HD costs	174 (1,342.4)	DP + HD costs	1,325 (3,276.3)
All services costs	11,162 (6,920.6)	All services costs	864 (2,892.5)	All services costs	6,920 (7,928.9)
Grand total costs	2,801,762	Grand total costs	1,467,256	Grand total costs	934,186

Stage at diagnosis is available for all cases in the initial phase of care only: 38% of cases are in stage I, 31% are in stage II, 23% in stage III and 8% in stage IV. Charlson Index is calculated for all patients having at least one hospital admission and the percentage of cases with undetermined Charlson Index varies by phase of care: 4, 55, 9% of cases in initial, continuing and the final phases, respectively.

All types of costs roughly follow a U-shape profile (Figure 3): hospitalisation costs profile has two peaks, in the first month after diagnosis (about 4,000 Euros) and in the last month of life (1,100 Euros); outpatient costs profile shows a peak in the 7th month of the initial phase and in the 3rd month before death; similarly for drugs costs profile.

As illustrated in Table 1, on yearly basis total cost per patient is higher among newly diagnosed patients (11,162 Euros per patient/year) and patients in the last year of life (6,920 Euros per patient/year). In the continuing phase the cost per patient/year is 864 Euros. There are differences in the composition of costs by health service across phases: among newly diagnosed patients, hospitalisation costs are highest (74% of total cost), followed by outpatient costs (18% of total cost) and drugs (8% of total cost). In the continuing phase, only 1/3 of costs are due to hospitalizations, 46% to outpatients and 20% to drugs. At the end of life, hospitalisation accounts to about a half (52%) of total cost, followed by 29% of outpatients and 19% of drugs. Overall costs of the entire study cohort of 2,085 colon cancer patients amount to about 2.8 million Euros in the initial, about 1.5 million Euros in the continuing and 934,000 Euros in the final phase of care.

We fit GLM to the yearly costs of patients in each phase of care separately, using the covariates described in Table 1, and compare the fit of two competing models: a linear regression model with normal errors (LM) and a Gamma regression model with log link function (GLM). The Akaike Information Criterion (AIC) (37) identifies the optimal model as the one minimising the value of the criterion. In order to identify the most appropriate set of covariates to explain the outcome of interest, we applied the backward selection procedure based on the AIC. The procedure was initialised with the full model, involving all the aforementioned variables, and sequentially dropped the potential predictors one at a time until the AIC was optimised, so that only covariates with a statistically significant explanatory power are actually considered as the determinants of the total annual costs. AIC minimum value is always associated to the GLM and is remarkably lower than the one obtained from the LM, endorsing the adoption of the Gamma assumption.

Table 2 reports the results of the fitting in each phase of care: AIC values of all models are reported in the upper part; followed by estimates of covariates, together with their *p*-value in the middle part; and finally R^2^ values are shown in the bottom part. All covariates are categorial, except “No. treatments”; if a categorical variable admits m categories, estimates of m-1 categories are compared to the reference category (indicated as “reference”). Estimates of covariates are reported only if they have at least one statistically significant category estimate; *p*-values < 0.05 are reported in bold.

**Table 2:** AIC values for the LM and Gamma-GLMs estimated on the total annual costs data, regression coefficient estimates for the optimal Gamma GLM and corresponding R^2^ values, by phase of care.

**Initial phase**			**Continuing phase**			**Final phase**		
**Model**	**AIC**		**Model**	**AIC**		**Model**	**AIC**	
LM	5,008.6		LM	29,023.0		LM	2,634.9	
GLM	4,899.0		GLM	22,565.5		GLM	2,465.5	
**Variable**	**Estimate**	***p*** **-value**	**Variable**	**Estimate**	***p*** **-value**	**Variable**	**Frequency**	***p*** **-value**
**Gender**			**Gender**			**Gender**		
Male	–	–	Male	Reference		Male	–	–
Female	–	–	Female	−0.285	**0.001**	Female	–	–
**Age class**			**Age class**			**Age class**	–	
[15,60]	–	–	[15,60]	0.180	0.210	[15,60]	0.372	0.249
[60,70]	–	–	[60,70]	Reference		[60,70]	reference	
[60,75]	–	–	[60,75]	0.120	0.371	[60,75]	−0.180	0.605
[75,85]	–	–	[75,85]	−0.182	0.104	[75,85]	−0.379	0.148
[85,110]	–	–	[85,110]	−0.214	0.174	[85,110]	−0.829	**0.005**
**Stage**			**Years since diagnosis**			**Years since diagnosis**		
I	Reference		[1,2]	Reference		[0,1]	−0.290	0.233
II	0.257	**<0.001**	[2,3]	0.025	0.887	[1,3]	reference	
III	0.51	**<0.001**	[3,4]	0.179	0.342	[3,4]	−0.332	0.307
IV	0.958	**<0.001**	[4,8]	0.021	0.896	[4,20]	−0.853	**<0.001**
			[8,20]	−0.274	0.077			
**Charlson Index**			**Charlson Index**			**Charlson Index**		
0	Reference		0	Reference		0	reference	
>0	−0.020	0.790	>0	0.940	**<0.001**	>0	−0.428	**0.019**
NA	−2.745	**<0.001**	NA	−0.710	**<0.001**	NA	−2.725	**<0.001**
No. treatments	0.002	**0.014**	No. treatments	0.028	**<0.001**	No. treatments	0.006	**<0.001**
**R** ^**2**^			**R** ^**2**^			**R** ^**2**^		
58%			51%			48%		

Values in bold are statistically significant.

As regards to the identification of the relevant predictors of the cost outcomes, one of the most interesting findings concerns the explanatory contribution of the Charlson Index. This covariate is statistically significant in all the models, but the sign of its association with the response depends on the disease phase: the economic impact of comorbidities is significantly increased with respect to the no-comorbidity status during the continuing phase, whereas it is significantly reduced during the final phase.

Focussing on the initial phase, our analysis confirms the significant effect of the stage at diagnosis: the higher the stage, the higher the costs. This evidence is testified by the increasing trend of the corresponding estimates of the regression coefficients and supports the crucial importance of an early cancer diagnosis, even from an economic point of view.

Additionally, in the final phase older patients and those with a cancer diagnosed at least 4 years before are associated with lower costs.

Finally, we stress that the R^2^ values of the estimated models range from 48 to 58%, indicating that the selected covariates in our regression analysis explain a notable portion of the original outcome variability, especially when compared to the regression approaches described in the literature where healthcare costs are modelled without accounting for the POC (31).

## Discussion

We illustrated the approach developed to tackle the economic burden of cancer issue in the framework of the Italian Epicost study. Main features of this approach are: (a) use of micro-data from multiple data sources (surveillance and administrative data); (b) use of a cross-sectional population-based study cohort; (c) identification of cancer-related procedures, treatments and drugs by a panel of experts; (d) phase of care framework to tackle the entire disease pathway; (e) detection of cost driving components *via* modelling.

The novelty of this approach is the development of a comprehensive framework spanning from data collection to the identification of main determinants of cancer cost, through the integration of a surveillance data source (the cancer registry) and administrative databases containing observed costs.

This approach has a number of strength points:

1. Thanks to data from high quality, comprehensive, population-based cancer registries, results can be interpreted at population level and may provide insights to policy makers for improving healthcare policies on cancer burden control.
2. A study based on micro-data enables the identification of clinical and demographic components that have greater influence on the distribution of costs.
3. A cross-sectional study design yields estimates of prevalence-based costs that provide an updated snapshot of current costs due to cancer treatments. This is particularly interesting for healthcare policy makers, who are typically interested in the optimal investment of the resources to guarantee the efficient coverage of the total expenses in 1 year. Moreover, this design allows to capture effects of technological changes, particularly relevant at disease onset and at the end of life, when main treatments are provided, and costs are higher. Finally, we use current cost data directly collected at constant price, hence there is no need for temporal price adjustment.
4. Differently from the incremental costs approach, that estimates only the extra cost due to cancer and requires a control cohort, the attribution approach identifies lists of cancer-related procedures and drugs, and allows to estimate direct cancer-related costs and to describe them in terms of patterns of care.
5. The POC framework enables to describe patients' patterns of care—from cancer onset to recovery/death—as well as to estimate costs along the patients' clinical pathway (costs profiles). This feature addresses the aim of the Europe's Beating Cancer Plan^3^ to tackle the entire disease pathway, with ten flagship initiatives and multiple supporting actions.
6. GLMs are applicable within the framework of Epicost to identify the main determinants of costs. A great variety of covariates (demographic, clinical and organisational) have been tested, and the most significant are: stage at diagnosis in the initial phase of care; presence of comorbidities in the continuing and final phases of care; older age at prevalence in the final phase of care. Some of the statistically significant covariates available in the Epicost dataset have never been earlier considered in the healthcare costs literature (37).
7. The model of analysis proposed here is replicable to other countries with different healthcare systems, as long as the cancer registry data can be linked to the administrative data, on an individual basis. Replicability has been assessed in other European countries, such as Belgium, Norway and Poland in the framework of the Innovative Partnership for Action Against Cancer (iPAAC) Joint Action, sponsored by the European Commission.^4^

On the other hand, the reader should be aware that costs data are collected for administrative, rather than surveillance purposes, and may be affected by quality and completeness issues. This is the case for Italy, where we experienced the following issues:

- hospital data are based on the Diagnosis-related group (DRG) coding system that does not allow to disentangle cost of one single treatment from another within the same hospitalisation event;
- diagnosis is not specified in outpatient and drugs data; even though we rely on cancer registry data for the identification of cancer cases, nevertheless a cancer diagnosis in the administrative data would help in the identification of cancer-related treatments and procedures;
- access to individual-based data is time consuming and requires dedicated resources, and this issue has been identified at European level as well, within the Join Action iPAAC: although in Europe there is a common General Data Protection Regulation (GDPR), access to data differs by country due to specific data protection features; moreover, quality and completeness of databases containing cost information are very different across countries, some of these differences depending on country specific healthcare delivery systems and policies; finally, interoperability of cancer information systems is limited by lack of common standards;
- Finally, unlike initial and final phases, where patients' patterns of care are more homogeneous, the continuing phase of care is made of a mixture of patients with different clinical characteristics and patterns of care: some patients are fully recovered; some others experience relapses; other patients live in chronic conditions. This variability is reflected in the large standard deviations of the corresponding mean costs (Table 1). On the basis of the information collected by CR, it is currently impossible to distinguish among these groups of patients, therefore costs estimated in the continuing phase reflect an average clinical condition and this might limit the interpretation of results. In order to address this limit, we included a comorbidity indicator, the Charlson Index, as covariate in the GLM. This variable however, although significantly related to costs, is missing for more than half of the continuing phase patients that experienced no hospitalizations. In the future, we intend to identify specific treatments and procedures targeted to specific patient categories and to use them to disentangle groups of patients with homogeneous care needs within the continuing phase of care (38, 39).

Possible extension of the Epicost approach will be directed towards projections by scenarios, in order to investigate the impact on the cost estimation of: (1) epidemiological changes in incidence and survival, due to screening or to the introduction of new diagnostic procedures, or new and more effective treatments; and (2) demographic changes due to population ageing.

Future developments on cancer costs evaluation in European countries are possibly related to the implementation of common procedures to access data according to the GDPR and to the sharing of methodologies to produce comparable results. Despite their complexity, international comparisons are fundamental to improve, generalise and extend cost evaluation to different contexts, and might provide further insights into cancer patient management and best practises to increase efficiency of healthcare delivery (40).

## Data availability statement

The raw data supporting the conclusions of this article will be made available by the authors, without undue reservation.

## Ethics statement

Ethical review and approval was not required for the study on human participants in accordance with the local legislation and institutional requirements. Written informed consent for participation was not required for this study in accordance with the national legislation and the institutional requirements.

## Author contributions

Material preparation, data collection, and analysis were performed by SG, GC, AG, CM, and SF. The first draft of the manuscript was written by SF and AG. All authors commented on previous versions of the manuscript, contributed to the study conception and design, read and approved the final manuscript.

## Funding

This study was funded by the Italian Ministry of Health (RICERCA FINALIZZATA 2018, Grant Number RF-2018-12365530) and by the European Commission (Work Programme 2017, Grant Number 801520 HP-JA-2017 “Innovative Partnership for Action Against Cancer”).

## Conflict of interest

The authors declare that the research was conducted in the absence of any commercial or financial relationships that could be construed as a potential conflict of interest.

## Publisher's note

All claims expressed in this article are solely those of the authors and do not necessarily represent those of their affiliated organizations, or those of the publisher, the editors and the reviewers. Any product that may be evaluated in this article, or claim that may be made by its manufacturer, is not guaranteed or endorsed by the publisher.

## Footnotes

^1^ http://www.epicost.it

^2^ https://www.ipaac.eu/en/work-packages/wp7

^3^ https://ec.europa.eu/info/strategy/priorities-2019-2024/promoting-our-european-way-life/european-health-union/cancer-plan-europe_en

^4^ https://www.ipaac.eu/en/work-packages/wp7/
